# Supplementary material for: Neurons along the auditory pathway exhibit a hierarchical organization of prediction error
Source: Nat Commun. 2017 Dec 15;8:2148. doi: 10.1038/s41467-017-02038-6 (PMC5732270; doi:10.1038/s41467-017-02038-6)
Supplement: Supplementary file 1 — Supplementary Information [file 41467_2017_2038_MOESM1_ESM.pdf]

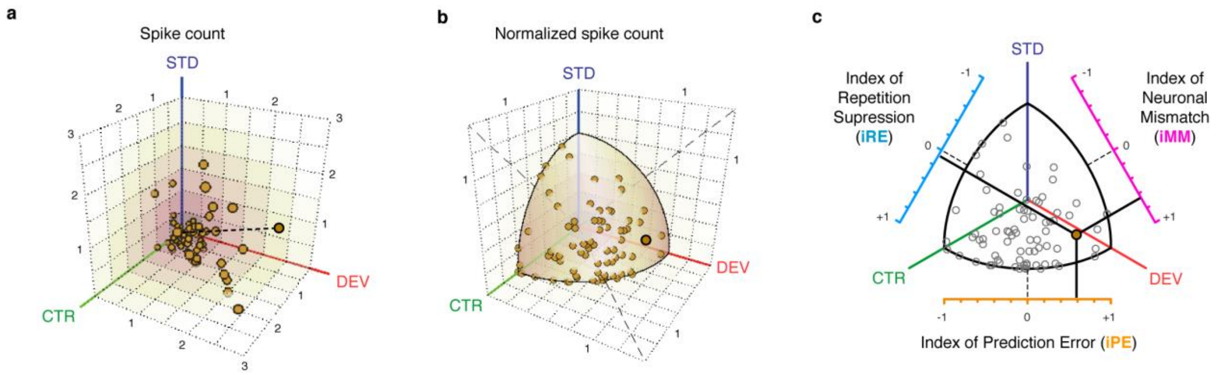

Supplementary Figure 1: Example of a population normalization procedure and representation. a. The response of each neuron to each tested tone recorded in deviant (DEV), standard (STD) and control (CTR) conditions defines a point in 3D space. **b.** Normalization of these three associated responses to a value between 0 and 1. Each point represented in (a) appears now radially projected onto a unit sphere centered on origin. **c.** Indexes result from the difference between two of these normalized responses, represented as color axes surrounding the scatter plots. The dotted black lines marks the absence of difference between conditions (index=0). Index values for each individual neuronal response (grey dots) can be consulted in the color axes as exemplified with the highlighted response (brown dot, solid black bar projecting from it to each index axis). The scatter plot is represented flattened from a zenith view of the unit sphere in order to simplify and facilitate overall legibility.

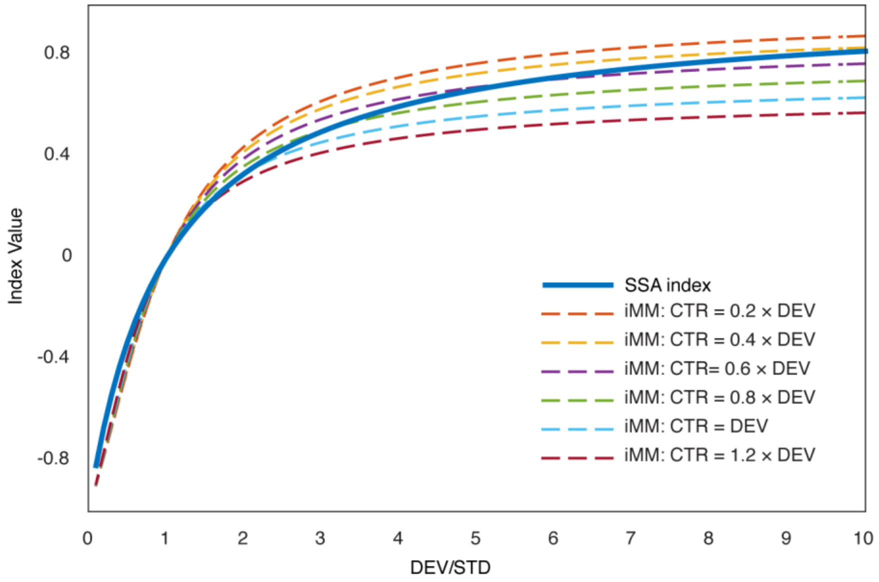

Supplementary Figure 2: Quantitative comparison between iMM and the "classical" SSA index. Quantitative comparison between iMM and the "classical" SSA index. The SSA index trace is plotted as a function of the DEV/STD ratio, since SSA does not take into account the control condition. Different iMM traces are plotted (dashed lines), as a function of the relative magnitude of the response to control condition with respect to deviant response (*CTR/DEV*), from low (*CTR=0.2*DEV*) to high (*CTR=1.2*DEV*) hypothetical responses to the control. Note that the two indices (the SSA index and the iMM for different CTR response magnitudes) tend to take values very close to each other.

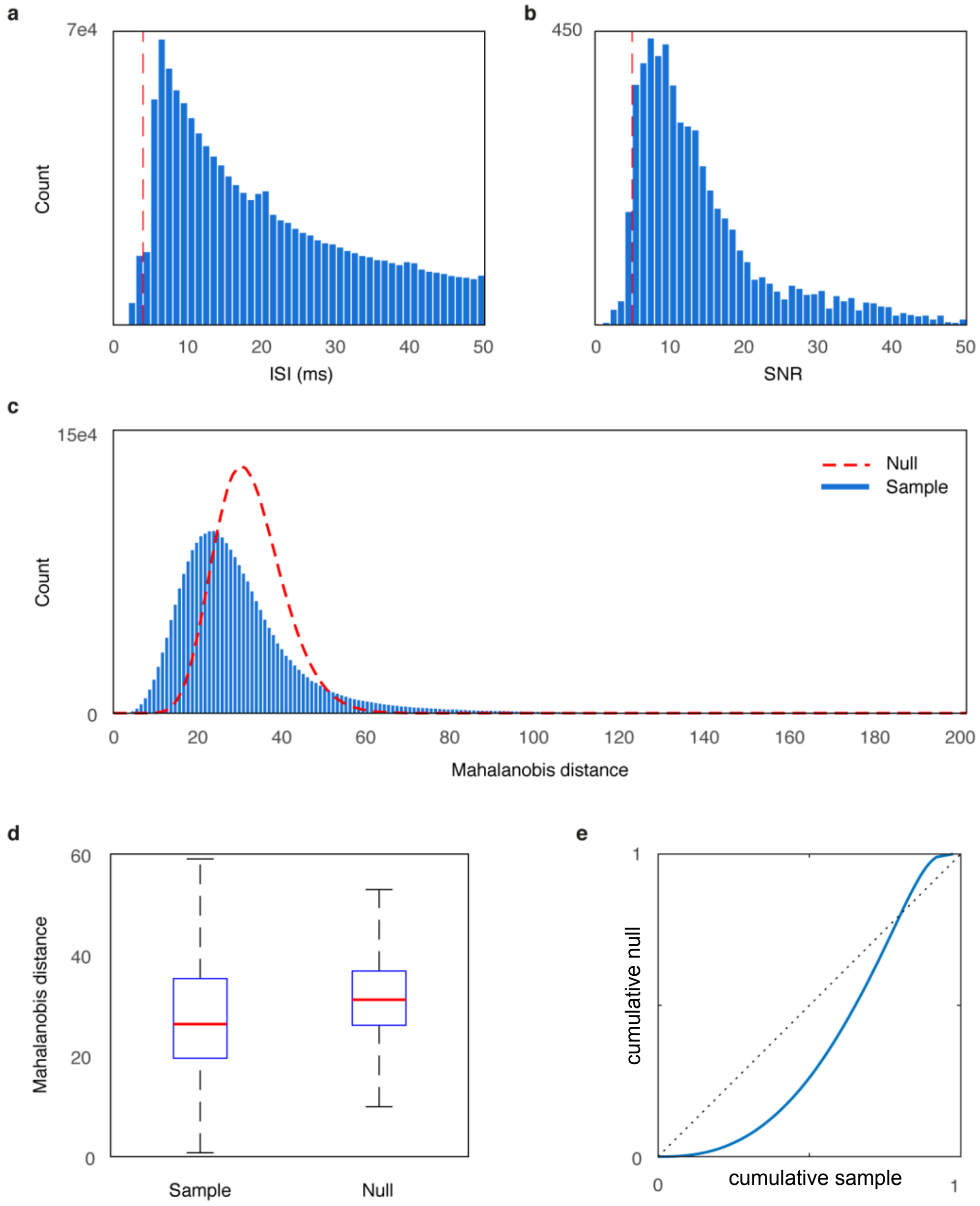

Supplementary Figure 3: Measures of spike isolation quality. a. Inter-spike interval (ISI) distribution for all our anesthetized recordings (2552731 individual spike waveforms from 5871 record files from all stations). For each individual spike, the time interval (in ms) to the previous spike is computed, and all these (2 million+) values are represented in the histogram. The long, thin tail beyond 50 ms is not shown for clarity, to show that less than 2% single spikes waveform occurred less than 4 ms (a reasonable refractory period) after the previous spike. **b.** Distribution of spike-amplitude-to-noise-ratio (SNR), for all sets of spikes waveform S recorded. More than 96% of our recorded spikes had at least 5 times more amplitude than the 2-3 std deviations set as threshold. **c.** Distribution of Mahalanobis distance for each single spikes waveform w recorded (blue histogram). If our spikes were purely normally distributed following a single 32-dimensional Gaussian distribution, the distribution of mahal (w, S) values for all spikes waveform w would look like the red dotted line. The real distribution is a left-skewed version of the former, indicating that our spikes waveform were even closer to each other in shape than in a standard single-spike cluster. **d.** This is confirmed by the boxplot comparative, showing that median and inter-quartile range were closer to zero

than the reference (null) distribution. **e.** As Mahalanobis distance d increases from 0 to +Inf, a point of the blue trace is defined as [cdf_sample(d), cdf_null(d)], where cdf is the cumulative density function of each distribution. Thus, the blue line compares all quantiles of these distributions, and shows that all quantiles of the sample distribution up to $Q_{0.8}$ correspond to lower quantiles of the reference distribution.

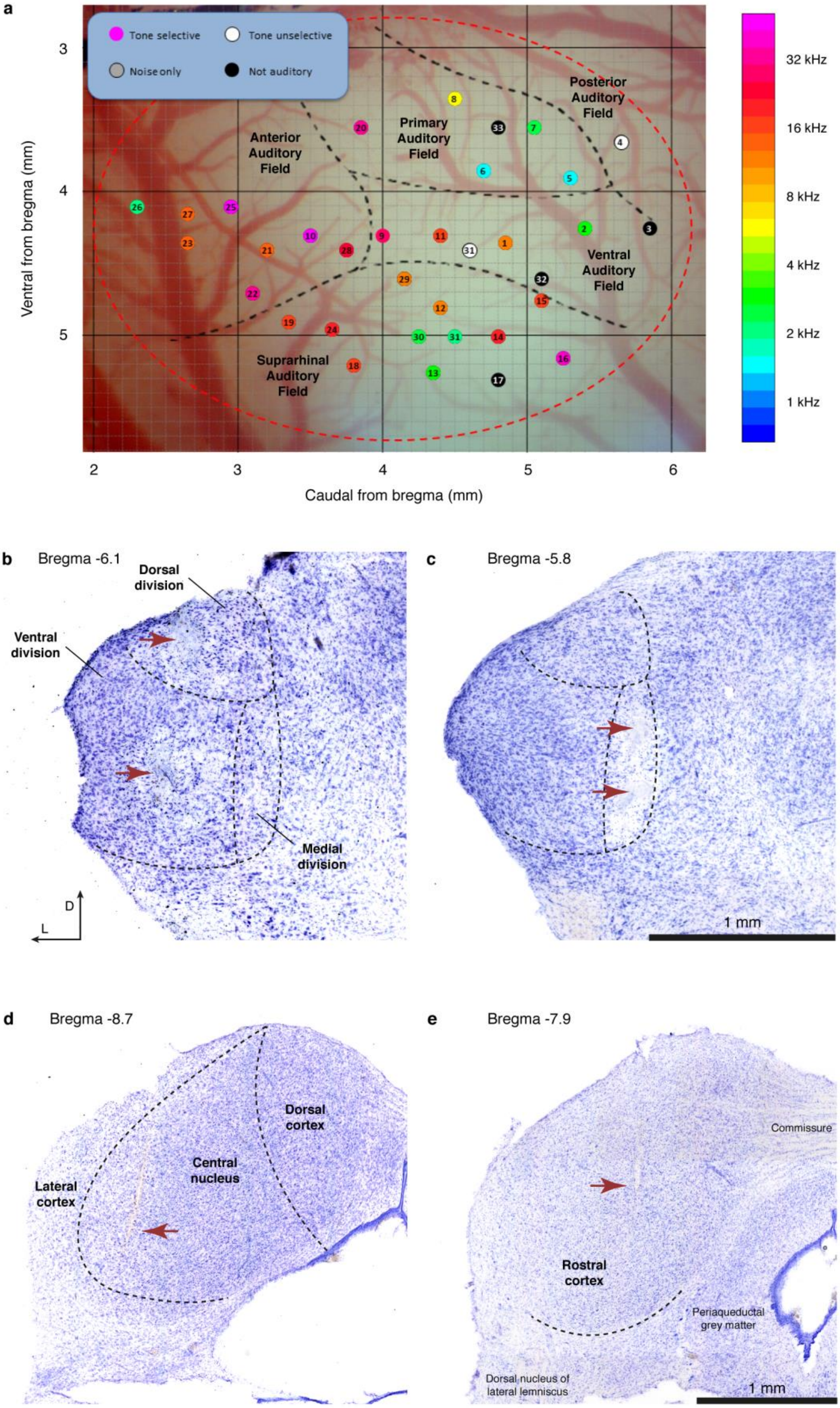
a
Tone selective
Tone unselective
Noise only
Not auditory
Anterior Auditory Field
Primary Auditory Field
Posterior Auditory Field
Ventral Auditory Field
Suprarhinal Auditory Field
32 kHz
16 kHz
8 kHz
4 kHz
2 kHz
1 kHz
Ventral from bregma (mm)
Caudal from bregma (mm)
b
Bregma -6.1
Dorsal division
Ventral division
Medial division
D
L
c
Bregma -5.8
1 mm
d
Bregma -8.7
Dorsal cortex
Central nucleus
Lateral cortex
e
Bregma -7.9
Commissure
Rostral cortex
Periaqueductal grey matter
Dorsal nucleus of lateral lemniscus
1 mm

Supplementary Figure 4: Anatomy for anesthetized rat preparations. a. Localization of all recordings made in the AC of one rat, in coordinates with respect to bregma. The characteristic frequency) of each track was determined (from all recordings made in that track, see Methods). Inversions of the characteristic frequency progression define the limits between cortical fields[1,2], so that all or most recordings can eventually be assigned to a particular field: primary, anterior, ventral, posterior or suprarhinal auditory field. **b.** Sample Nissl-stained histological slice showing electrolytic lesions along one electrode track (red arrows). Applying depth interpolation, all recordings made in that track could be assigned to either ventral or dorsal divisions of the MGB[3]. **c.** Same as in (b), showing lesions along a track that traverses the medial division of the MGB[3]. **d.** In this case, the whole electrode track through the central nucleus of the inferior colliculus can be clearly seen up to the point where the lesion was made (red arrow; lateral cortex of the IC[4,5]). **e.** Sample of a lesion marking a neuron recorded in the rostral cortex of the IC[4,5]). Scale bars: 1 mm in each case.

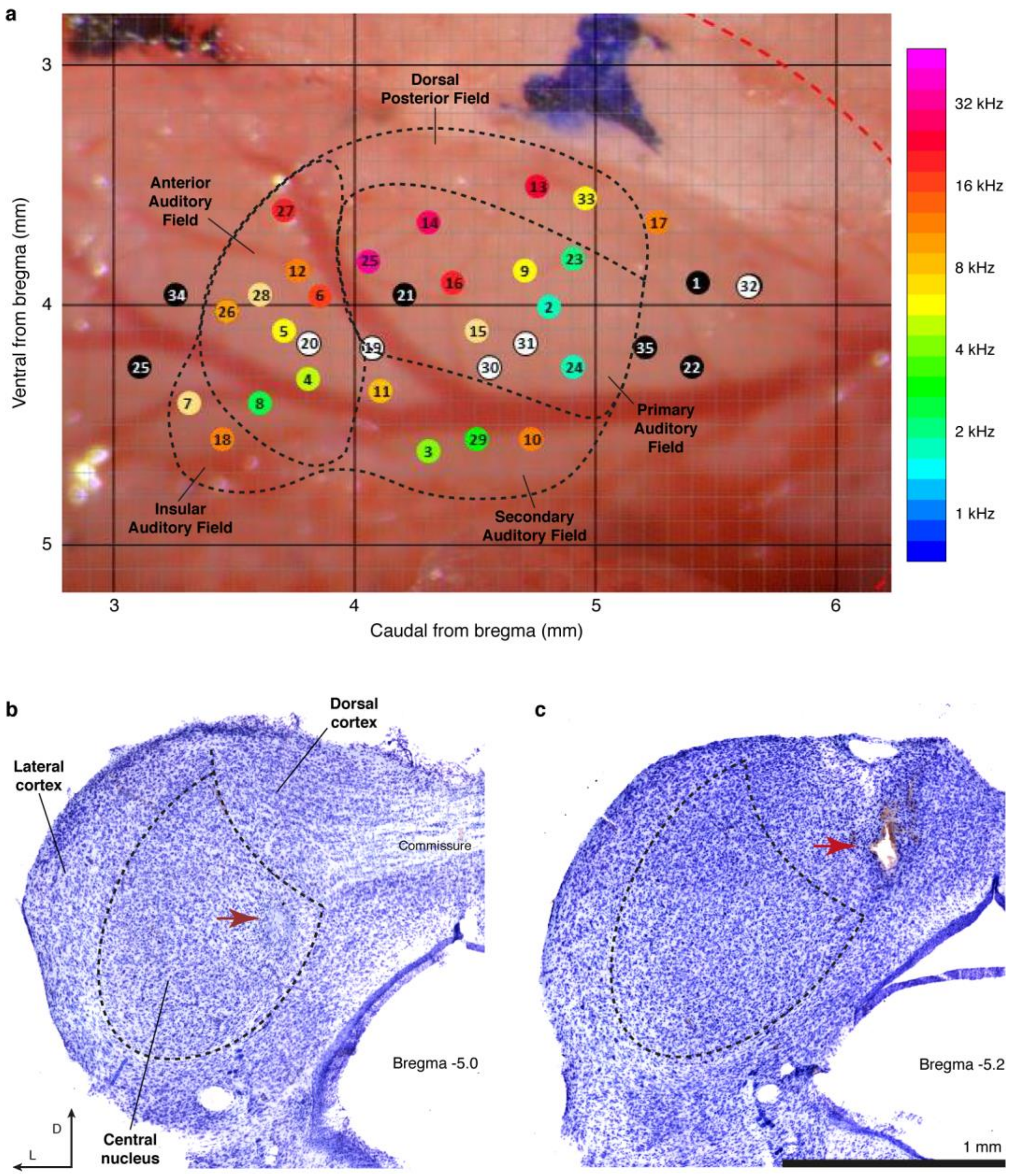

Supplementary Figure 5: Anatomy for awake mice. a. Localization of all recordings made in the AC of one mouse, in coordinates with respect to bregma. The characteristic frequency of each track was determined (from all recordings made in that track, see Methods). Inversions of the characteristic frequency progression define the limits between cortical fields[6,7], so that all or most recordings can eventually be assigned to a particular field: primary, secondary, anterior, dorsal posterior or insular auditory field. **b, c.** Sample Nissl-stained histological slice showing an electrolytic lesion (red arrow) in central nucleus in one case (b) or dorsal cortex in a different mouse (c). Scale bar: 1 mm.

Supplementary Table 1: List of abbreviations

Abbrebiation	Definition
AC	Auditory Cortex
AC_L	Lemniscal areas of the Auditory Cortex
AC_{NL}	Non-lemnical areas of the Auditory Cortex
CTR	Control
DEV	Deviant
FDR	False Discovery Rate
IC	Inferior Colliculus
ICL	Lemniscal region of the Inferior Colliculus
ICNL	Non-lemniscal regions of the Inferior Colliculus
iMM	Index of Neuronal Mismatch.
iPE	Index of Prediction Error
iRS	Index of Repetition Supression
L	Lemniscal pathway
LFP	Local Field Potential
MGB	Medial Geniculate Body
MGB_L	Lemniscal regions of the Medial Genicualte Body
MGB_{NL}	Non-Lemniscal regions of the Medial Geniculate Body
MMN	Mismatch Negativity
NL	Non-Lemniscal pathway
PE-LFP	Prediction error potential
SPL	Sound Pressure Level
SSA	Stimulus-Specific Adaptation
STD	Standard

Supplementary References:

1. Nieto-Diego, J. & Malmierca, M. S. Topographic Distribution of Stimulus-Specific Adaptation across Auditory Cortical Fields in the Anesthetized Rat. PLoS Biol. 14, 1–30 (2016).
2. Paxinos, G. & Watson, C. The Rat Brain in Stereotaxic Coordinates. (Elsevier Science, 2013).
3. Antunes, F. M., Nelken, I., Covey, E. & Malmierca, M. S. Stimulus-specific adaptation in the auditory thalamus of the anesthetized rat. *PLoS One* **5,** e14071 (2010).
4. Malmierca, M. S., Blackstad, T. W. & Osen, K. K. Computer-assisted 3-D reconstructions of Golgi-impregnated neurons in the cortical regions of the inferior colliculus of rat. *Hear. Res.* **274,** 13–26 (2011).
5. Loftus, W. C., Malmierca, M. S., Bishop, D. C. & Oliver, D. L. The cytoarchitecture of the inferior colliculus revisited: A common organization of the lateral cortex in rat and cat. *Neuroscience* **154,** 196–205 (2008)
6. Joachimsthaler, B., Uhlmann, M., Miller, F., Ehret, G. & Kurt, S. Quantitative analysis of neuronal response properties in primary and higher-order auditory cortical fields of awake house mice (Mus musculus). Eur. J. Neurosci. 39, 904–918 (2014).
7. Polley, D. B., Read, H. L., Storace, D. A. & Merzenich, M. M. Multiparametric Auditory Receptive Field Organization Across Five Cortical Fields in the Albino Rat. J. Neurophysiol. 97, 3621–3638 (2007).
